# Maintaining differential pressure gradients does not increase safety inside modern BSL-4 laboratories

**DOI:** 10.3389/fbioe.2022.953675

**Published:** 2022-08-30

**Authors:** Andreas Kurth, Udo Weber, Detlef Reichenbacher

**Affiliations:** ^1^ Biosafety Level-4 Laboratory, Robert Koch Institute, Centre for Biological Threats and Special Pathogens, Berlin, Germany; ^2^ Ingenieurbüro Udo Weber, Köln, Germany; ^3^ Construction, Physical Plant and Technology, Robert Koch Institute, Central Services, Berlin, Germany

**Keywords:** BSL-4 laboratory, differential pressure, directional airflow, biosafety, maximum containment laboratory, risk analysis (assessment)

## Abstract

This article discusses a previously unrecognized contradiction in the design of biosafety level-4 (BSL-4) suit laboratories, also known as maximum or high containment laboratories. For decades, it is suggested that both directional airflow and pressure differentials are essential safety measures to prevent the release of pathogens into the environment and to avoid cross-contamination between laboratory rooms. Despite the absence of an existing evidence-based risk analyses demonstrating increased safety by directional airflow and pressure differentials in BSL-4 laboratories, they were anchored in various national regulations. Currently, the construction and operation of BSL-4 laboratories are subject to rigorous quality and technical requirements including airtight containment. Over time, BSL-4 laboratories evolved to enormously complex technical infrastructures. With the aim to counterbalance this development towards technical simplification while still maintaining maximum safety, we provide a detailed risk analysis by calculating pathogen mitigation in maximum contamination scenarios. The results presented and discussed herein, indicate that both directional airflow or a differential pressure gradient in airtight rooms within a secondary BSL-4 containment do not increase biosafety, and are not necessary. Likewise, reduction of pressure zones from the outside into the secondary containment may also provide sufficient environmental protection. We encourage laboratory design professionals to consider technical simplification and policymakers to adapt corresponding legislation and regulations surrounding directional airflow and pressure differentials for technically airtight BSL-4 laboratories.

## Introduction

Handling and working with human pathogens, depending on their classification of risk groups 1–4, takes place in laboratories of respective biosafety levels 1–4 (BSL-1 to BSL-4). To reliably prevent cross-contamination of samples and exposure of employees and the environment, numerous safety measures are used in laboratories of the highest biosafety level 4 (BSL-4), also described as maximum containment laboratory (MCL), which have been developed and established over the past decades in step with the state-of-the-art in science and technology.

**FIGURE 1:**
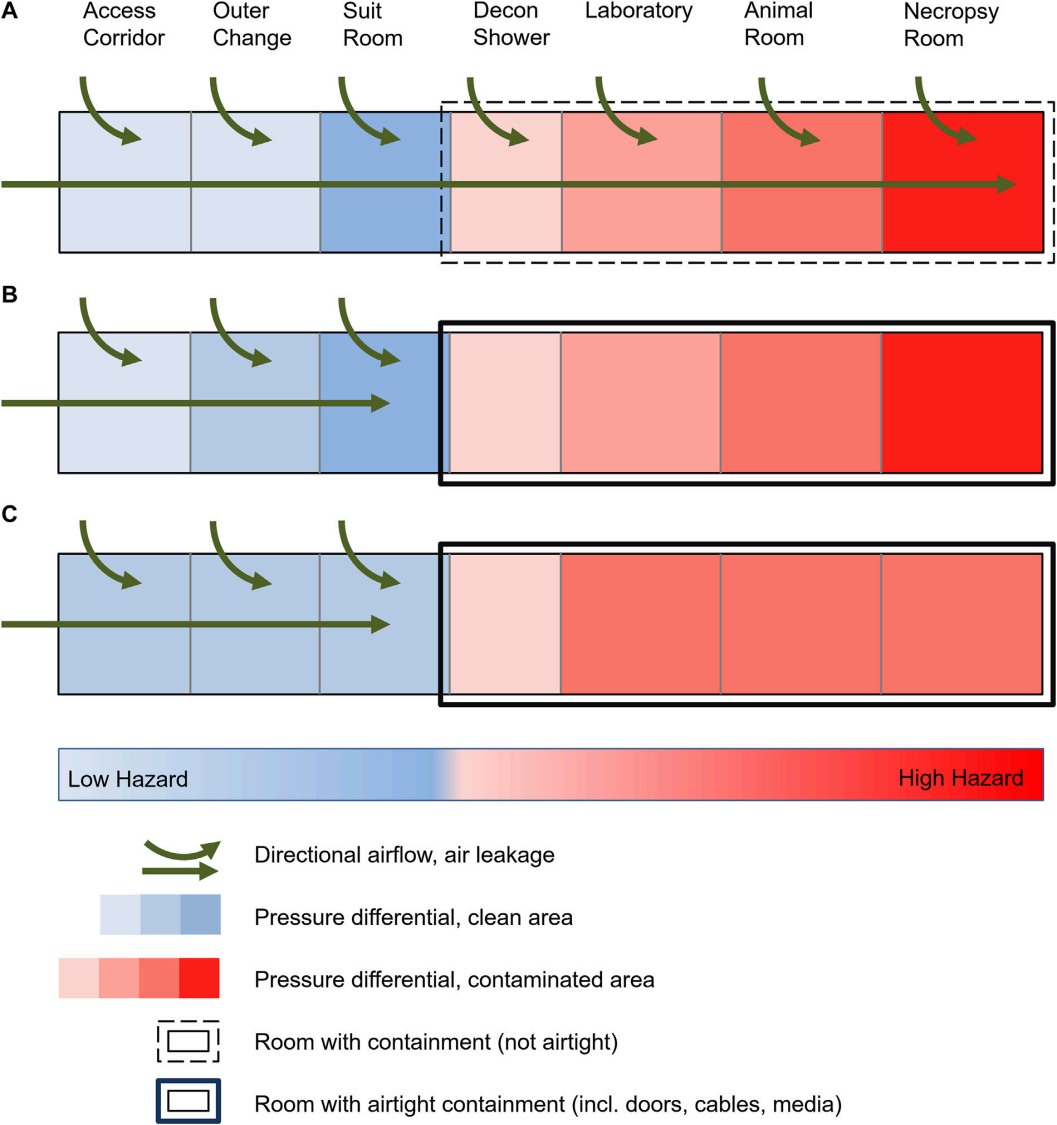
Schematic of a high-security laboratory with targeted air flow and pressure differentials. **(A)** Historical laboratories with common leakage due to standard construction practices, targeted air flow and pressure levels up to the area of greatest contamination. **(B)** Modern laboratories with technically airtight containment and maintenance of pressure levels up to the area of greatest contamination. **(C)** Risk assessment-based reduction in the number of pressure levels in a BSL-4 laboratory with airtight containment despite unchanged protection against cross-contamination and protection of the environment.

In general, two different types of BSL-4 laboratories have been developed: cabinet laboratories and protective-suit laboratories. In Germany, laboratories were built exclusively for use with protective suits. For this purpose, in the second half of the 20th century, the parallel technical development of positive-pressure suits to protect laboratory workers and biosafety cabinets (BSC) to prevent sample cross-contamination were used. To prevent the release of pathogens into the environment, both a directional airflow by constant (negative) pressure differentials was set up via room ventilation systems between the outside and inside areas of the laboratory (Figure 1A), as well as effective filtration of potentially contaminated exhaust air from the laboratory into the outside environment. The entirety of these measures has been implemented as a safety standard worldwide and included in the relevant recommendations and regulations (LBG, 1996; National Research Council Committee on Hazardous Biological Substances in the Laboratory, 1989; Biosafety in microbiological and biomedical laboratories, 1999). The aim of the directional airflow and the pressure differentials was to prevent the escape of potentially contaminated air from the laboratory through any structural leaks of the containment barrier between the laboratory and the outside (e.g., through doors, walls, floors, roofs, pipelines). In the course of the following decades, technical advancements allowing for tighter structures and thus also of MCLs have been employed, which enable the generation and monitoring of controlled and constant air flow and differential pressure gradients between adjacent rooms. Of note, these advancements also resulted in an increased technical complexity, and dramatically increased construction, operation and maintenance expenses.

Contemporarily, the planning, construction and operation of BSL-4 laboratories are subject to very rigorous quality and technical requirements. To prevent the release of human pathogens to the external environment, high-efficiency particulate air (HEPA) filters are used to filter exhaust air from BSL-3 and BSL-4 laboratories, and the laboratories are operated at a constant negative pressure. The requirements for air tightness of the laboratories also increase with increasing biosafety levels. In addition to all other technical requirements and safety measures, a defined, technically airtight and appropriately monitored containment is an absolute necessity for BSL-4 laboratories consistently worldwide (Figure 1B) (Canadian Biosafety Standard, 2015; Biosafety in microbiological and biomedical laboratories, 2020). The requirements of the air tightness criteria (e.g., the generally accepted “Canadian Biosafety Standards and Guidelines”) are extremely high and therefore the air leakage volume is correspondingly small, while the air exchange rates within the rooms are maintained as high as possible. The advantage of an airtight and appropriately tested containment is, among other things, the increased protection of the environment, also in the event of a failure of the ventilation system or the occurrence of possible positive-pressure situations within the containment caused by technical faults. In Germany, this development led to the present legislation and regulations (BioStoffV 2013; Technische Regeln für Biologische Arbeitsstoffe, 2013; GenTSV, 2019), stating that access to the BSL-4 laboratory must traverse four airlocks (outer change room, personal hygienic shower, suit room, decontamination shower) with a differential pressure gradient. Furthermore, the established principle of directional airflow and pressure differentials is also requested within the laboratory depending on the contamination risk and was established from areas with potentially lower contamination risk to areas with highest contamination, e.g., from the main laboratory to animal rooms to necropsy rooms (Figure 1B). Similar requirements have been established worldwide (for text excerpts see Supplementary Table S1). However, an experimentally or computationally determined basis for evaluating the risk of potential exposure to biomaterials under normal operation or accident situations in a BSL-4 laboratory, animal holding or necropsy rooms are not considered in any of the regulations cited. Such a risk analysis of the alleged increased safety by directional airflow and the pressure differentials has not yet been published since the beginning of the operation of BSL-4 laboratories.

The technical implementation of differential pressure gradients between technically airtight rooms for BSL-4 laboratories, required by current regulations, is achieved by a specifically adjusted and controlled ratio of supply and exhaust air for each individual room. The air exchange rate per room of 12–15 times per hour ensures the dilution and removal of air contaminated by infectious microorganisms via the exhaust air through downstream HEPA filtration. For additional protection of staff within the laboratory, it is required that infectious material be processed only under a BSC (or comparable equipment), regarded as primary containment, while staff wear a ventilated positive-pressure protective suit. Following the successful technical implementation of defined, airtight containments for BSL-4 laboratories, the benefit or necessity of directional airflow and pressure differentials has not been evaluated to date. Due to the lack of experimental data, international and national microbiological guidelines do not suggest levels of negative pressures and the levels of pressure differentials required to effectively prevent cross-contamination with risk group 4 pathogens. Also, there are no data for the potential safety impact of changing the level of pressure differentials on potential cross-contamination. Only a 2005 study by (Bennett et al.,2005) addresses the relationship between negative pressure and protection from cross-contamination in BSL-3 laboratories in an evidence-based manner and concludes that pressure differentials has no effect on protection from cross-contamination. Only directional airflow into a laboratory (inflow velocity) had a positive effect and is still used today to protect against cross-contamination in non-technically airtight rooms (e.g., BSL-3 laboratories).

The currently established and practiced differential pressure gradients are operated within the technical limits of the available measurement, control and regulation technology and have no empirical or biological basis. Worldwide, pressure differences of 30–60 Pa between adjacent rooms are used in BSL-4 laboratories, depending on technical possibilities. Both the actuating forces of doors in existing negative pressure cascades must be controllable and the air pressure controls for adjacent rooms (Δp) must have a sufficient limit distance from each other to avoid pressure disturbances. If the aforementioned “Canadian Biosafety Standards and Guidelines” for the tightness of the containment are complied with, the remaining air leakage is no longer relevant in this respect and is to be disregarded. Consequently, the question arises as to whether a reduction in the target directional airflow and the pressure differentials would result in an increased risk of contamination, which in turn raises the question of the extent to which a target directional airflow and the differential pressure gradients between technically airtight rooms fundamentally contributes to a reduction in the risk of contamination.

Considering the technical development of BSL-4 laboratories, we discuss in this article whether a directional airflow and/or differential pressure gradients are still necessary to minimize a contamination risk. To do this, we calculate the likelihood of contamination within a room and its spread to adjacent rooms, considering leakage volumes in airtight rooms, pressure equalization when a sealed door is opened, and the “displacement” of air when a person passes through a door.

## Basis and calculations

The BSL-4 laboratory at the Robert Koch Institute, Berlin, Germany, was used as the basis for the following calculations. The laboratory was built according to the air tightness criteria of the Canadian Guideline (Canadian Biosafety Standard, 2015) and has been in regular operation since 2018 after construction completion in 2015.

Access to the laboratory rooms is via four airlocks with differential pressure gradients (−20 Pa, −40 Pa, −80 Pa [suit room], −120 Pa [decontamination shower]). In this process, the negative pressures in the respective airlocks increase towards the laboratory rooms and are thus intended to protect the environment by targeted directional airflow from the outside to the inside. Within the laboratory, further differential pressure gradients are applied to the areas with the highest probable risk of contamination (−160 Pa [cell culture], −200 Pa [animal room], −240 Pa [necropsy room]). The determination of the differential pressure gradient values followed the national and international regulations for BSL-4 laboratories and were planned for in 2008 (laboratory planning) with no separate, specially prepared risk analysis. The pressure differentials were designed to allow the actuating forces of the doors to be manageable in existing differential pressure gradients and also to allow Δp controls for the rooms to have a sufficient limit distance from each other to avoid pressure disturbances.

To our knowledge, no data have been published about the quantity of generated infectious bioaerosols during normal BSL-4 laboratory operation or accident situations in a cell culture laboratory, animal room, or necropsy room. Furthermore, it is comprehensible that bioaerosol generation during animal husbandry depends on the animal model or infection model and the caging systems used. It is also comprehensible, that working with infectious viruses under a BSC, handling animals in individually ventilated cages (IVC) and changing stations, or performing a necropsy on a downdraft table, considering their protection factor, would generate less bioaerosols than an accidental release of virus in a room, e.g., while dropping and breakage of a sample flask or vial. Therefore, for the risk assessment presented herein, we evaluate a worst-case practical scenario of contamination in a BSL-4 laboratory, using experimental data with spores from (Bennett and Parks, 2006), as well as a constant hypothetical generation of bioaerosols during an animal experiment in conventional cages. Standard and accepted fluid mechanics and thermodynamics formulas were used for all calculations.

The study by (Bennett and Parks, 2006) describes a single release of biomaterials in a defined room during various laboratory accidents. The dropping of a sample vessel (50 ml) with a spore suspension of 2 × 10^9^ spores/ml (total of 1 × 10^11^ spores) in an 18 m³ room was investigated as the scenario of the highest potential for contamination, and an aerosol release of 1.03 × 10^3^ spores/m³ (in relation to the room dimension, a total of 1.9 × 10^4^ spores) was measured. To simulate a comparable laboratory accident in the BSL-4 laboratory, the release of a maximum possible virus concentration in the laboratory was considered. The scenario assumed here is the dropping of a sample vessel (50 ml) with a virus concentration of 2 × 10^8^ viruses/ml (total 1 × 10^10^ viruses) in the laboratory. This amount corresponds to the maximum of viruses per volume processed in the BSL-4 laboratory at the Robert Koch Institute. According to the ratio of the release measured by (Bennett et al.,2005) a total of approximately 2 × 10^3^ viruses would be released as aerosols in a room. The remainder of the virus-containing suspension would remain surface bound and would be removed immediately after dropping by decontamination of the affected surfaces. All calculations made here are performed with the assumption of a maximum bioaerosol release of 2 × 10^3^ viruses. Since a fully equipped laboratory filled with furniture will most likely not provide a situation for an optimal release and distribution of bioaerosol as performed by (Bennett and Parks, 2006), we believe the assumed maximum release of 2 × 10^3^ viruses is rather exaggerated, but already considers an added margin of potential error.

For evaluation of a continuous contamination by infected animals, an extensive literature search was conducted. Despite robust evidence supporting the airborne transmission, and hence bioaerosol release, of many respiratory viruses, including measles virus, influenza virus, respiratory syncytial virus, human rhinovirus, adenovirus, enterovirus, severe acute respiratory syndrome coronavirus (SARS-CoV), Middle East respiratory syndrome coronavirus (MERS-CoV), SARS-CoV-2, and Zaire Ebola virus (Weingartl et al., 2012; Wang et al., 2021), very limited data are published about the quantitative release of airborne pathogens. Of those, the majority of air samples are analyzed for the presence of viral genome copy numbers, which do not indicate the quantity of infectious virus. Some extrapolations from genome copy numbers to infectious particles have been presented in various aerosol study, ranging from 10:1 to as much as 10^5^:1 (Hawks et al., 2021; Tellier, 2022), indicating the unreliability of such extrapolations. Direct infectious virus quantification from air samples was performed in a study of SARS-CoV-2 (concentration between 6 and 74 TCID_50_ per liter air) in a hospital room with two COVID-19 patients (Lednicky et al., 2020), in an experimental infection study of Syrian hamsters with SARS-CoV-2 with an average emission rate per animal of 25 infectious virions/hour on days 1 and 2 post inoculation (Hawks et al., 2021), and experimental infection studies of ferrets with influenza virus H1N1 with average emission rates per animal of <4 and 11 PFU/min (Gustin et al., 2013) and 7 to 138 PFU/min (Singanayagam et al., 2020). For the risk assessment presented herein, the hypothetical virus-containing bioaerosol release is calculated for the maximum number of the largest animal used in commercially available conventional cages (without primary containment) with a polyester filter sheet (TECNIPLAST 2000P) at the BSL-4 laboratory at the Robert Koch Institute: 48 infectious adult guinea pigs (e.g., as a possible animal model for human disease) with an average emission rate of 100 viruses/minute. The calculations estimating possible aerosol and virus release are given in the text below.

### Very low leakage volume in airtight rooms

To protect the environment, modern BSL-4 laboratories are built with airtight rooms (walls, doors and penetrations) that allow the lowest possible leakage. To calculate the leakage rate of a sample room with 60 m³, the tightness requirement is based on the pressure drop method according to the recognized Canadian guideline (at 500 Pa negative pressure, this may drop max. to −250 Pa within 20 min).

Definitions:

LW Air exchange rate.

V̇_Zu_ Supply airflow.

V̇_Ab_ Exhaust airflow.

V_Ri_ Room volume.

P_A_ Low pressure at the start.

P_E_ Low pressure at the end.

V_A_ Initial volume at low pressure at the start.

V_E_ Final volume at low pressure

dV̇ Leakage volume.

The sample room of V_Ri_ = 60 m³ has an airflow of 900 m³/h at LW = 15 1/h.

$${\dot{V}}_{\mathrm{Zu}}\;/\;{\dot{V}}_{\mathrm{Ab}}\;=900\frac{m^{3}}{h}$$

According to Canadian guideline, at 500 Pa negative pressure and a maximum drop to −250 Pa within 20 min corresponds:

P_A_ = regular air pressure 100,000 −500 Pa = 99,500 Pa.

P_E_ = regular air pressure 100,000 −250 Pa = 99,750 Pa.

The allowable leakage rate at constant temperature and atmospheric pressure is given by the equations:

$$\frac{V_{A}}{V_{E}}=\frac{P_{\mathrm{AE}}}{P_{A}}$$

As well as

$$V_{E}=V_{A}-\mathrm{dV}\;\left(\text{Formula by Boyle Mariotte}\right)$$

After conversion and merging, the following formula is obtained for the leakage volume:

$$V_{E}=\;\frac{P_{A\cdot \;}V_{A}}{P_{E}}$$

 PA  ·   VA PE=VA−dV

$$\mathrm{dV}=\;V_{A}-\;\frac{P_{A}\cdot V_{A}}{P_{E}}$$

dV=−  PA  ·   VA PE+VA

$$\mathrm{dV}=-\;60\;\cdot \;\;\frac{99,500\;}{99,750}+60=0.15m^{3}³$$

This results in a leakage airflow/h with closed doors of:

60 min/h 20 min⋅0.15 m3=0.45 m3/h

For a sample room of 60 m³, the allowable leakage rate is 0.45 m³/h (0.75%/h). Following the Canadian guideline, the sample room would have a very low leakage volume and a leakage rate under operation of less than 0.45 m³/h. Therefore, within a BSL-4 laboratory with airtight doors, no directional airflow is applicable. The airflow controllers used for the individual rooms have a deviation of ± 5 % (45 m³/h at an airflow of 900 m³/h) and thus a deviation too large to accurately evaluate the room tightness. Therefore, a corresponding pressure test is carried out annually.

### Immediate pressure equalization when opening an airtight door

**FIGURE 2:**
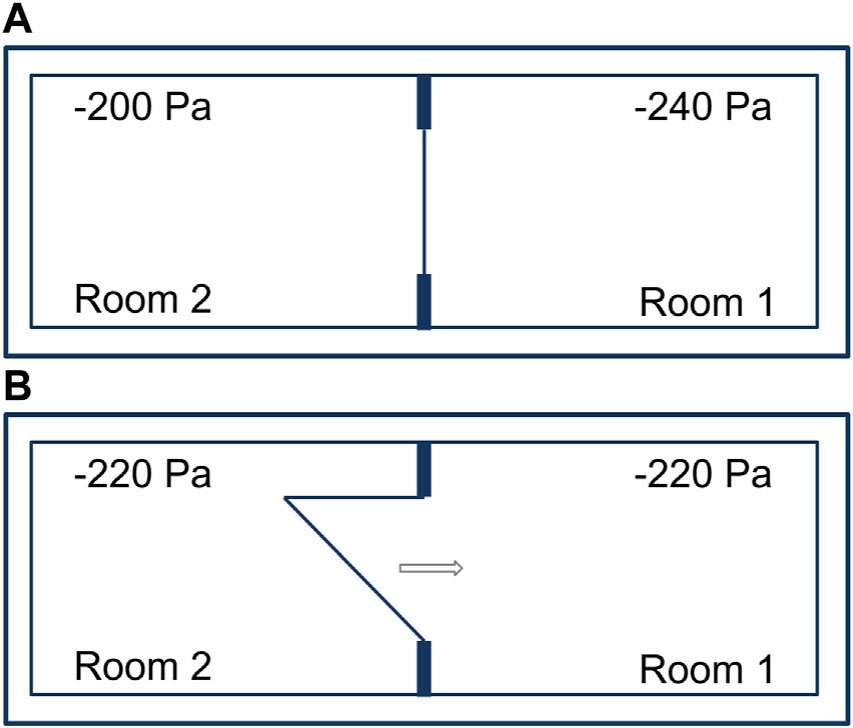
Adjacent laboratory rooms within a technically airtight BSL-4 laboratory. **(A)** Separated by an airtight door and operated with a pressure differential of 40 Pa. **(B)** Immediate pressure equalization when opening an airtight door.

Adjacent laboratory rooms separated by an airtight door and operated with a pressure differential of 40 Pa (Figure 2A). Opening an airtight door inside a BSL-4 laboratory results in pressure control circuits being activated. The control circuits of the two neighboring rooms with pressure differentials oscillate and lead to irrational, uncontrollable pressure fluctuations. To avoid this, when a door is opened, both control loops of the rooms are “frozen” for the time the door is open, i.e., the controllers remain in the control position that existed before the door was opened and do not resume operation until the door is sealed. The amount of supply and exhaust air thus remains the same in both rooms during door actuation. After opening a door and interrupting the control function, there is inevitably a rapid pressure equalization between the two rooms, occurring in less than a second (Figure 2B). This involves extremely low air volumes of 0.3% or 0.4 %, depending on the rooms, relative to the total volume of the two rooms. Therefore, during the time of door opening, no directional airflow is applicable. For a pressure difference of 40 Pa between two rooms (−200 Pa and −240 Pa), the volume for pressure equalization is calculated as follows:

P_A_ = regular air pressure 100,000 −240 Pa = 99,760 Pa.

P_E_ = regular air pressure 100,000 −200 Pa = 99,800 Pa

$$\mathrm{dV}=\;60-\;\frac{99,760}{99,800}\;\cdot \;60=0.024m^{3}³$$

When a door from a 60 m³ room is opened, 0.024 m³ of air flows from laboratory room 2 (−200 Pa) to laboratory room 1 (−240 Pa). Pressure equalization takes place immediately and even before the door is open wide enough for a person to pass through.

### Person “dragging” air when passing through a door

**FIGURE 3:**
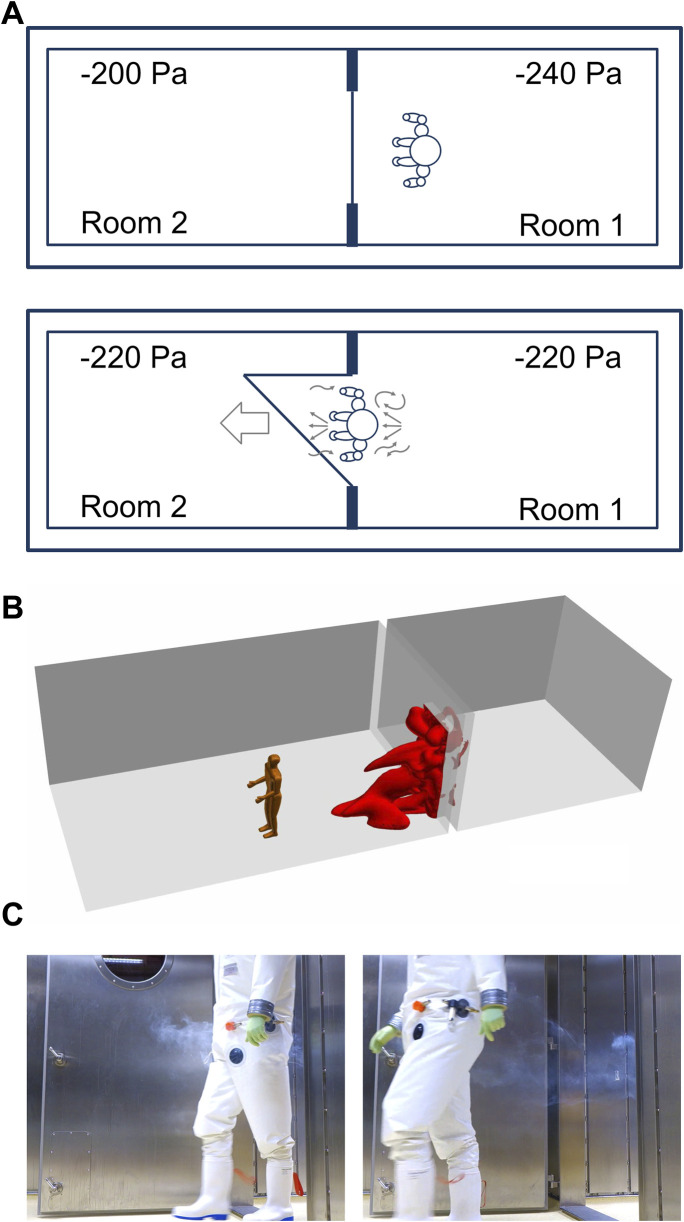
A Person is walking between adjacent laboratory rooms within a technically airtight BSL-4 laboratory. **(A)** Person “dragging” air when passing through a doorway. **(B)** Simulation of air dragged into a room 15 s after a person is passing through a doorway. **(C)** Illustration of dragged air by a person walking from room 1 into room 2 distributing smoke before the person began to walk.

When passing a doorway from laboratory room 1 to laboratory room 2, a person is dragging approximately 0.76 m³ of air (Figures 3A–C). The air volume was calculated by a computational fluid dynamics (CFD) simulation (Figure 3B). For this purpose, a model was chosen in which a person with a height of 1.80 m is moved through a door (door size 2.1 m × 1.1 m) at a speed of 1 m/s (3.6 km/h). After 15 s the door is closed.

The dragged air volume caused by the person is 32 times greater than the air movement caused by pressure equalization (calculated above) and can also occur in a direction opposite to the airflow caused by the pressure equalization in response to a door opening. Likewise, the movement of the person through the door will cause a small increase in pressure in laboratory room 2, resulting in the backflow of air into laboratory room 1 until the pressure is equalized again.

### Virus distribution after contamination within a room

Two different scenarios were considered for the risk assessment in the event of the release of a maximum possible virus concentration in the BSL-4 laboratory. The virus distribution is calculated in scenario A for the case of a laboratory accident and in scenario B for the case of animal husbandry in conventional cages without primary containment. The influence of homogeneous distribution in the room as well as air exchange is considered.

In scenario A, the release of bioaerosols containing a total of 2 × 10³ viruses in a room of 60 m³ is assumed after the breakage of a sample vial on the floor (50 ml virus suspension). If a sample of 50 ml breaks, only a portion of the virus is resultantly aerosolized. The largest portion wets the floor or other surfaces. Droplets sink back to the ground after breakage, a portion sticks to surfaces, another portion floats in the air (actual aerosols). After 10 min, an approximately homogeneous distribution of the suspended aerosols in the room can be assumed. The air exchange rate is 15 times/h. To dispose of the broken sample vessel and decontaminate the site following standard operating BSL-4 procedures, the person remains in the room for at least 15 min without opening any door. Within 15 min, 225 m³ of air is exchanged. To calculate the remaining number of virus particles in bioaerosols in the room, the formula for recovery time equation (Raatz and Luftwechsel, 2006) was used:

$$C_{\mathrm{NT}}=C_{N\infty }+\left(C_{\mathrm{NO}}-C_{N\infty }\right)e^{\mathrm{-\beta}\cdot \varepsilon \cdot t}$$

*β* = 0.25 1/min (Air exchange rate).

*Ɛ* = 0.8 (Ventilation efficiency).

*t* = 15 min.

C_NT_ = Current particle concentration.

C_NO_ = 34 viruses/m³ (2 × 10^3^ viruses released in 60 m³).

C_N∞_ = 0 (Estimated final concentration)

$$C_{\mathrm{NT}}=0+\left(\mathrm{34}-0\right)e^{\mathrm{-0}\mathrm{.25}\cdot 0\mathrm{.8}\cdot \mathrm{15}}$$

= **34 · 0.0498**.

= **2 viruses/m³**

After breaking a sample vial on the floor and waiting for 15 min, there are approximately 2 virus particle/m³ as bioaerosols in the room.

In scenario B, the hypothetical virus distribution is calculated for the case of a large animal husbandry situation of 48 adult guinea pigs (occupancy with 12 cages of four animals each as a possible animal model for human diseases) in conventional cages with polyester filter sheet cover. For lack of data on aerosol excretion of risk group-4 pathogens in experimental animals, a value of 100 viruses/minute/animal, comparable to SARS-CoV-2 in hamsters or influenza virus in ferrets (see details above), is hypothesized for the following calculations. In this hypothetical respiratory infection, 48 adult guinea pigs would exhale 2.9 × 10^5^ viruses as bioaerosol within 1 h. The aerosol reduction by the polyester filter sheet of approximately 92% (TECNIPLAST Conventional Cages), 2.3 × 10^4^ viruses are released into the room per hour. The dilution in the room of 60 m³ results in a release per hour of 384 viruses/m³.

To calculate the virus concentration after homogeneous distribution at an assumed maximum released quantity of 384 viruses/h/m³ in the laboratory with 60 m³ and an air exchange rate of 900 m³/h follows:

$$C_{\mathrm{NT}}=C_{N\infty }+\left(C_{\mathrm{NO}}-C_{N\infty }\right)e^{\mathrm{-\beta}\cdot \varepsilon \cdot t}$$

C_NT_ = Concentration after homogeneous distribution, current particle concentration.

C_NO_ = Input, original particle concentration

*β* = 0.25 1/min (Air exchange rate).

*Ɛ* = 0.8 (Ventilation efficiency).

t = 20 min (Time span for safe homogeneous distribution)

$$C_{\mathrm{NT}}=0+\left(\mathrm{384}-0\right)e^{\mathrm{-0}\mathrm{.25}\cdot 0\mathrm{.8}\cdot \mathrm{20}}\left(\text{Value for permanent input}\right)$$

= **384 · 0.0183**.

= **7.0 viruses/m³**

Considering the 15 air exchanges per hour, it can be assumed that a virus load in bioaerosols in the room caused by conventional animal caging will remain comparable to the accidental release of bioaerosols from scenario A. In the case of open animal housing and fleece paper, the concentration is reduced to below 7 viruses/m³ for the duration of maximum bioaerosol excretion.

### Spread of viruses to adjacent rooms

First, the influence of the opening time of an airtight door connecting 2 neighboring laboratory rooms is discussed. It is to be noted that the door opening time has practically no influence on the entrainment of air (and aerosols), since the entrainment is decisively influenced exclusively by the movement of a person or objects through the doorway. The minimal air exchange (see calculations under 2.2), which occurs in case of existing pressure differentials between rooms, has no significant entrainment effect and is physically absent in case of connecting rooms with equal pressure. Also, the room pressure condition is not affected by room temperature if the negative pressure control per room is well adjusted, even for small and common room temperature differences. As already stated above, no directional airflow is applicable between individual airtight rooms.

First, we consider the influence of waiting time (5, 10, and 20 min) on virus concentration after maximum room contamination (scenario A) before a door to an adjacent room is opened (Figure 2A). When a person (0.76 m³) passes through a doorway from room 1 to room 2 (Figure 3A), 0.76 m³ of air in rooms without a pressure differential, or 0.736 m³ (0.76 m³—0.024 m³) of air in rooms with pressure differential is carried over.

$$\mathrm{a)}\;C_{\mathrm{NT}}=C_{N\infty }+\left(C_{\mathrm{NO}}-C_{N\infty }\right)e^{\mathrm{-0}\mathrm{.25}\cdot 0\mathrm{.7}\cdot 5}$$

= 0 + (34—0) e ^−0.25^ ^· 0.7 · 5^

= 14.3 viruses/m³ (after a 5 min waiting time).

= 0.4 viruses displaced by pressure equalization only (with pressure differential).

= 10.5 viruses displaced by a person (with pressure differential).

= 10.9 viruses displaced by a person (without pressure differential).

Of note: with 5 min of waiting time, an uneven distribution in the room is assumed (0.7 instead of 0.8)

$$\mathrm{b)}\;C_{\mathrm{NT}}=C_{N\infty }+\left(C_{\mathrm{NO}}-C_{N\infty }\right)e^{\mathrm{-0}\mathrm{.25}\cdot 0\mathrm{.8}\cdot \mathrm{10}}$$

= 4.6 viruses/m³ (after a 10 min waiting time)

$$\mathrm{c)}\;C_{\mathrm{NT}}=C_{N\infty }+\left(C_{\mathrm{NO}}-C_{N\infty }\right)e^{\mathrm{-0}\mathrm{.25}\cdot 0\mathrm{.8}\cdot \mathrm{20}}$$

= 0.6 viruses/m³ (after a 20 min waiting time).

A waiting time after a virus contamination leads to a reduction of the virus load due to the air exchange, whereby the absolute virus load of 14 viruses/m³ after 5 min is negligibly low despite a maximum release within the containment. After a maximum release of virus and opening of an airtight door, no virus (0.4) would move into the other room despite a pressure differential of 40 Pa. The amount of air dragged by a person, and therefore potential virus cross-contamination (11 viruses) is also negligible and does not differ in the presence or absence of pressure differentials between adjacent rooms. With a maximum virus load of 7 viruses/m³ during animal experiments, the same insignificant risk for cross-contamination into the adjacent room can therefore be expected.

## Discussion

When the first BSL-4 laboratories were built in the 1960-80s, necessary structural safety barriers were established to prevent pathogens from escaping the laboratories. These included an individual supply and exhaust air system that could create pressure differentials and directional airflow to prevent contamination from areas within the laboratory with the highest potential risk toward areas outside the laboratory. Accordingly, the directional airflow gradient was established from the area of lowest exposure risk to the area of highest exposure risk to biosubstances (outside area → decontamination shower → laboratory → animal holding; Figure 1A). This was assumed necessary to avoid contamination of the environment or cross-contamination to adjacent laboratory rooms due to the technological air leakage of the laboratories. With the development of airtight doors and sealed pipelines, laboratories with increasing airtightness could be built starting in the 1980s. Today, this is state-of-the-art, with the degree of airtightness being high and evaluated annually. However, the necessity and usefulness of the originally required pressure differentials and a directional airflow gradient has not been questioned or re-evaluated. The relevant requirements remain unchanged in national and international guidelines in this respect (Technische Regeln für Biologische Arbeitsstoffe, 2013; BioStoffV 2013; Canadian Biosafety Standard, 2015; GenTSV 2019; Biosafety in microbiological and biomedical laboratories, 2020). Only the most recent version of the WHO Laboratory Biosafety Manual (Laboratory design and maintenance, 2020) re-evaluated the strict determination of risk groups and biosafety levels, instead encouraging the evidence-based and transparent assessment of the risks to allow safety measures to be balanced with the actual risk. It is stated, that controlled pressure differentials should be designed for a MCL from the least to the most contaminated area when necessary, indicating possible unspecified scenarios when pressure differentials might not be necessary or even useful.

Due to the construction of airtight rooms, which results in a very low leakage volume of no more than 0.75% of the room volume per hour with the doors sealed, the benefit of a directional airflow is insignificant to the maintained air exchange rate. When a door is opened, only a very brief, negligible directional airflow occurs into an adjacent room with a pressure gradient (1/30th of what is caused by a person traversing a doorway). Thus, directional airflow loses its intended benefit of preventing cross-contamination into adjacent laboratory spaces. This means that pressure differentials between airtight rooms within containment does not reduce the risk of aerosol carryover. Therefore, passive air exchange with open doors or air displaced by people and moving objects are the sole factors to consider for possible cross-contamination into adjacent laboratory rooms.

During normal operation of a BSL-4 laboratory, the use of primary containment (safety cabinets, downdraft tables with filtered air exhausts, IVC cages for animal containment, or animal changing stations) reliably prevent significant contamination within a room. The use of positive-pressure suits provides further protection for laboratory personnel. The additional high air exchange rates ensure a contamination-free laboratory area. Cross-contamination is only conceivable in special situations (e.g., release of virus-containing samples outside the safety cabinet, animal husbandry without primary containment, failure of the ventilation system). To our knowledge, there are no data on cross-contamination in BSL-4 laboratories, although our theoretical calculations suggest that such contamination would not be measurable. In general, the amounts of viral material processed in a BSL-4 laboratory are very low. Hence, the maximum amount of bioaerosols released during an accident (2 × 10^3^ viruses from a of total 1 × 10^10^ viruses in a vessel) implies a small biosafety risk, compared to situations in clinical settings. It is therefore not surprising, that even after release of the largest possible amount of virus by breaking a sample vial and a waiting time of 20 min, our mathematical model shows no bioaerosol presence (arithmetically 0.6 viruses/m³ in a 60 m³ room) due to the high air exchange rate. Even in the most unfavorable case of a maximum release without a waiting time, the number of aerosol-contained viruses (arithmetically 34 viruses/m³ in a 60 m³ room) is too low for a possible contamination of adjacent laboratory rooms. The theoretical calculations in this study clearly shows that there is no difference of the contamination risk into adjacent laboratory rooms with open doors with or without pressure differentials, even after the maximum release of viruses and only 5 min of waiting time (arithmetically 0 versus 0.4 virus from a 60 m³ room) or by air displacement by a person (arithmetically 10.5 versus 10.9 viruses/m³ after 5 min of waiting time in a 60 m³ room). A change in the hazard potential could arise, for example, when processing large quantities of virus or using large animals with a correspondingly high aerosol release. A detailed risk assessment for any individual BSL-4 laboratory should be carried out to evaluate the level of protection of laboratory personnel and the environment before requiring directional airflow and pressure differentials.

As a result of the above investigation and calculations, pressure differentials outside of the secondary containment areas remain necessary. A pressure differential in the decontamination shower as the outer secondary containment boundary and transition to the inner containment spaces are justified and reasonable in the event of a door leakage. In contrast, differential pressure gradients in entrance airlocks do not represent an additional increase in safety. As a logical consequence, the authors consider a total of three pressure levels to be sufficient if it can be excluded that the suit room could be potentially contaminated (e.g., by overriding the door in an emergency). This could be guaranteed if the door from the decontamination shower to the suit room can only be opened after complete decontamination (with a shortened decontamination cycle, implying no emergency egress). This would have to be substantiated by a risk assessment of the respective user. If this question cannot be answered unambiguously and clearly, a further, fourth pressure level is required. This results in a minimal 3-zone differential pressure gradient (possibly four zones), which represents a significant reduction of current practice (Figure 1C): access corridor/outer change/suit room → decontamination shower → laboratory (possibly corridor/changing room → suit room). In principle, in the authors’ opinion, a fixed number of pressure levels in legal regulations or ordinances is not practical and does not add to safety. The necessity and usefulness of the number of pressure levels depending on laboratory operation should always be assessed, proven and confirmed on the basis of risk assessments.

Considering the calculations presented above and the risk assessments carried out, the following conclusions can be drawn for the operation of the BSL-4 laboratory at the Robert Koch Institute, and likely apply to other BSL-4 laboratories throughout the world:

1) Due to the airtight construction with airtight doors and sealed pipelines, there is no actual directional airflow within the containment facility; not even when a door is opened.
2) An accidental release of a virus-containing sample outside a biosafety cabinet (e.g., dropping/brakeage of a cell culture vessel) represents the situation for the highest room contamination.
3) Regardless of the animal model and virus, an animal holding with primary containment (IVC) has no increased room contamination potential compared to normal laboratory operation.
4) Depending on the animal model and virus, animal husbandry in conventional cages without primary containment most likely results in lower or similar room contamination than point 2.
5) By processing animals individually in the necropsy room using a downdraft table with filtered exhaust air, room contamination is comparable to normal laboratory operation and lower than point 2.
6) Due to the low residual bioaerosol contamination of a maximum of 14 viruses/m³ after the highest possible room contamination and a waiting time of 5 min or during an animal experiment using conventional cages, the air displacement of a person (including a maximum of 10 viruses), the risk of cross-contamination to an adjacent laboratory room is negligible.
7) The risk of bioaerosol movement from an area with potentially higher contamination to areas with lower contamination is insignificant due to the low virus concentrations and limited air displacement.
8) An increase of biosafety risk by potential contamination due to the elimination of pressure differentials within a secondary containment with airtight doors is excluded.
9) A uniform pressure level within the secondary containment including laboratory, animal room and necropsy room does not increase the safety risk.
10) Since contamination in the suit room is excluded, three pressure levels (suit room, decontamination shower, laboratory) provide a sufficient environmental protection.

## Conclusion

An attempted directional airflow between technically airtight spaces does not contribute to reducing the risk of cross-contamination due to the very low leakage volume. Thus, directional airflow or a differential pressure gradient in airtight rooms within a secondary containment area do not increase biosafety and are no longer necessary. The only decisive biosafety factor is sufficient tightness of the secondary containment and the unconditional maintenance of the prescribed air exchange.

This simplifies the necessary operation and monitoring technology and workflows when the pressure differentials within the secondary containment are eliminated. At the same time, the simplified use of the laboratory increases occupational safety for the personnel working in the containment. Also, the control and regulation processes for controlling the pressure conditions of the secondary containment are simplified, and complex, highly sophisticated technical solutions for error-free door opening and closing are no longer required. This significantly reduces the probability of failure and significantly increases the availability and passive safety of these laboratories. Following the same rationale, a reduction of pressure levels from the outside into the secondary containment may also provide a sufficient environmental protection.

Adaptation of the legislation and regulations should occur for directional airflow and pressure differentials for technically airtight BSL-4 laboratories.

## Data Availability

The original contributions presented in the study are included in the article/Supplementary Material, further inquiries can be directed to the corresponding author.
