# Supplementary material for: Maintaining differential pressure gradients does not increase safety inside modern BSL-4 laboratories
Source: Front Bioeng Biotechnol. 2022 Aug 30;10:953675. doi: 10.3389/fbioe.2022.953675 (PMC9468669; doi:10.3389/fbioe.2022.953675)
Supplement: Supplementary file 1 [file Table1.DOCX]

Supplementary Material

# Supplementary Table S1. Text excerpts from legislations and guidelines.

| **Reference** | **Containment (sealed or gastight/airtight)** | **Differential pressure / directional airflow** |
| --- | --- | --- |
| *North America* | | |
| (1)  BL 1989 | The laboratory's distinguishing characteristic is that it has secondary barriers to prevent hazardous materials from escaping into the environment. Such barriers include sealed openings into the laboratory, airlocks or liquid disinfectant barriers, …  Walls, floors, and ceilings of the facility are constructed to form a sealed internal shell which facilitates fumigation... All penetrations in these structures and surfaces are sealed.  Entry to this area is through an airlock fitted with airtight doors.  All penetrations into the internal shell of the suit area are sealed. | An individual supply and exhaust air ventilation system is provided. The system maintains pressure differentials and directional airflow as required to assure flows inward from areas outside of the facility toward areas of highest potential risk within the facility.  The air pressure within the suit area is lower than that of any adjacent area. |
| (2)  Crane 1999 | There is not full agreement on the level of airtightness required in this shell. Some laboratories (USAMRIID, Lyon) do not pressure decay test the shell while others (Canada, Southwest) go through elaborate procedures to ensure a high degree of airtightness. Is the time and money required to create an absolute barrier necessary when the high differential directional air flow keeps contaminated air inside the facility? This is more debatable when you note that we purposefully pump air out of these facilities through double-banks of HEPA filters. | No specific pressure differential is required by BMBL and setpoints vary widely between laboratories. The bottom line is providing a pressure differential that will create sufficient directional inward airflow. |
| (3)  BMBL 1999 | Entry to this area is through an airlock fitted with airtight doors.  Walls, floors, and ceilings of the suit area are con- structed to form a sealed internal shell, …  All penetrations into the internal shell of the suit area, chemical shower, and airlocks, are sealed.  All penetrations in these structures and surfaces are sealed. | The supply and exhaust components of the system are balanced to ensure directional airflow from the area of least hazard to the area(s) of greatest potential hazard.  The differential pressure/directional airflow between adjacent areas is monitored and alarmed to indicate malfunction of the system. |
| (4)  BMBL 2020 | Entry into the laboratory is through an airlock fitted with airtight doors.  Walls, floors, and ceilings of the laboratory are constructed to form a sealed internal shell to facilitate fumigation and prohibit animal and insect intrusion. Floors are monolithic, sealed, and coved.  All penetrations in the internal shell of the laboratory, suit storage room, and the inner change room are sealed.  Atmospheric venting systems are provided with two HEPA filters in series and are sealed up to the second filter.  Windows must be break-resistant and sealed. | The ventilation system is designed to maintain the laboratory at negative pressure to surrounding areas and provide differential pressure or directional airflow as appropriate between adjacent areas within the laboratory. |
| (5)  LBG 1996 | Entry to laboratory via sealed air-lock.  Air supply and exhaust ductwork sealed airtight.  Equipped with bubble tight damper to permit sealing for decontamination procedures. | Directional inward, non-recirculated airflow  Rooms in isolation area to be maintained at pressure negative to corridor with greatest negative pressure in most hazardous room. |
| (6)  CBS 2015 | All penetrations of the containment barrier, including all conduits and wiring, to be sealed with a non-shrinking sealant that is compatible with the disinfectant(s) in use. Airtight doors to be provided for entry, including:  • the chemical decontamination shower doors;  • the inner and outer doors of the anteroom(s) dedicated to the entry of animals and equipment into the containment zone; and  • any critical door directly on the containment barrier.  Integrity of containment barrier to be tested by pressure decay testing. Acceptance criteria include two consecutive tests with a maximum of 250 Pa (i.e., 1 in. w.g.) loss of pressure from an initial 500 Pa (i.e., 2 in. w.g.) over a 20 minute period. | Inward directional airflow (IDA), established by HVAC system design through negative air pressure differentials, ensures that air flows from areas of lower containment or low risk of contamination to areas of higher containment (i.e., higher risk of contamination), never the reverse. This prevents the release and the spread of contamination to lower levels of containment by establishing a physical containment barrier of air against airborne or aerosolized infectious material or toxins. For example, IDA forces air to flow from the “clean” change area into the “dirty” change area, from the “dirty” change area into the laboratory work area, and from the laboratory work area into the animal cubicle.  There is a long-established principle of differential pressure to reduce potential cross-contamination within a BSL-4 laboratory by adjusting airflow from areas of potential lower contamination to highest contamination, i.e., main lab to animal areas to autoclave areas. |
| (7)  LBM 2020 |  | The ventilation systems must be designed to maintain controlled pressure differentials.  If necessary, controlled pressure differentials should be designed from the least to the most contaminated area. |
| (8)  LBM-LDM 2020 |  | The supply and exhaust components of the ventilating system are balanced to provide directional airflow within the suit area from the area of least risk to the area(s) of greatest risk.  All critical ventilation, pressure differential, life safety and operational systems must be continually monitored and have alarms. |
| *Germany National* | | |
| (9)  BioStoffV 2013 | It must be possible to seal the area with an assigned protection level for fumigation. | Permanent negative pressure shall be maintained in the area with an assigned protection level. |
| (10)  TRBA 100 2013 | The protection level area comprises one or more laboratories, as well as a four-chamber airlock system as an entrance and exit.  The doors of the airlock system must be sufficiently airtight so that there is no possibility of biological agents escaping.  All points at which supply and disposal lines pass through must be sealed and secured against backflow. | The protection level area must have a controlled, graduated negative pressure that increases from the airlock chambers to the working area in order to prevent air escaping from this area. |
| (11)  GenTSV 1990 | If windows are present, they must be airtight, unbreakable and unopenable. [Sind Fenster vorhanden, müssen sie dicht, bruchsicher und dürfen nicht zu öffnen sein.]  Entry to the laboratory must be through a three-chamber airlock only. [Die Arbeitsräume des Labors dürfen nur durch eine dreikammerige Schleuse betreten werden können.]  The walls, ceilings and floors of the laboratory must be sealed to the outside. All openings for service pipes must be sealed. [Wände, Decken und Fußböden des Labors müssen nach außen dicht sein. Alle Durchtritte von Ver- und Entsorgungsleitungen müssen abgedichtet sein.] | There must be a pressure differential between the airlock and the work rooms to prevent air from escaping from the isolated laboratory section. [Die Schleuse muss gegen den Vorraum und die Arbeitsräume mit einer entsprechenden Druckstaffelung versehen sein, um ein Austritt von Luft aus dem isolierten Laborteil zu verhindern.] |
| (12)  GenTSV 2019 | If windows are present, they must be airtight, unbreakable and unopenable. Preferably, visual links from the laboratory to the outside should be present and be made of airtight and unbreakable material. Entry to the laboratory must be through a four-chamber airlock only.  The walls, ceilings and floors of the laboratory must be sealed to the outside. All openings for service pipes must be sealed.  The supply and exhaust air ducts and the laboratory itself must be gastight and permit fumigation. | There must be a pressure differential between the airlock and the work rooms to prevent air from escaping from the isolated laboratory section.  The ventilation system shall be designed in such a way as to maintain a controlled negative pressure, relative to the outside world, in the laboratory at all times. The negative pressure must increase incrementally from the chambers of the airlock towards the work room. |

**References**

1. National Research Council (US) Committee on Hazardous Biological Substances in the Laboratory. Biosafety In The Laboratory (**BL**): Prudent Practices for the Handling and Disposal of Infectious Materials. Washington (DC): National Academies Press (US); 1989
2. Crane JT, Bullock FC, Richmond JY. Designing the BSL4 Laboratory. Richmond JY (eds.), Anthology of Biosafety: Perspectives on laboratory design. American Biological Safety Association, Journal of the American Biological Safety Association, 4(1) pp. 24-32; 1999
3. Biosafety in microbiological and biomedical laboratories (**BMBL**). 4th Edition. U.S. Dept. of Health and Human Services. HHS Publication No. (CDC) 99-xxxx, 1999
4. Biosafety in microbiological and biomedical laboratories (**BMBL**). 6th Edition. U.S. Dept. of Health and Human Services. HHS Publication No. (CDC) 300859, 2020
5. Laboratory Biosafety Guidelines (**LBG**), 2nd Edition. Ottawa: Minister of Supply and Services Canada, 1996
6. Canadian Biosafety Standard (**CBS**), Second Edition. Her Majesty the Queen in Right of Canada, as represented by the Minister of Health and the Minister of Agriculture and Agri-Food, 2015
7. World Health Organization. Laboratory biosafety manual (**LBM**). Fourth edition. Geneva: World Health Organization; 2020
8. Laboratory design and maintenance (**LBM-LDM**). Geneva: World Health Organization; 2020 (Laboratory biosafety manual, fourth edition and associated monographs)
9. Verordnung über Sicherheit und Gesundheitsschutz bei Tätigkeiten mit biologischen Arbeitsstoffen (Biostoffverordnung – **BioStoffV**) [Ordinance on safety and health protection at work involving biological agents (Biological Agents Ordinance)], 2013
10. Technische Regeln für Biologische Arbeitsstoffe: Schutzmaßnahmen für Tätigkeiten mit biologischen Arbeitsstoffen in Laboratorien (**TRBA 100**) [Technical Rules for Biological Agents: Protective measures for activities involving biological agents in laboratories]. Committee for Biological Agents (ABAS), 2013
11. Verordnung über die Sicherheitsstufen und Sicherheitsmaßnahmen bei gentechnischen Arbeiten in gentechnischen Anlagen (Gentechnik-Sicherheitsverordnung - **GenTSV**) [Ordinance on the Restructuring of the Law on the Safety Levels and Safety Measures for Genetic Engineering Operations in Genetic Engineering Installations]; 1990
12. Verordnung über die Sicherheitsstufen und Sicherheitsmaßnahmen bei gentechnischen Arbeiten in gentechnischen Anlagen (Gentechnik-Sicherheitsverordnung - **GenTSV**) [Ordinance on the Restructuring of the Law on the Safety Levels and Safety Measures for Genetic Engineering Operations in Genetic Engineering Installations]; 2019
